# Cannabinoid Receptors in the Central Nervous System: Their Signaling and Roles in Disease

**DOI:** 10.3389/fncel.2016.00294

**Published:** 2017-01-04

**Authors:** Debra A. Kendall, Guillermo A. Yudowski

**Affiliations:** ^1^Department of Pharmaceutical Sciences, University of ConnecticutStorrs, CT, USA; ^2^Department of Anatomy and Neurobiology, University of Puerto Rico, Medical Sciences CampusSan Juan, Puerto Rico; ^3^Institute of Neurobiology, University of Puerto RicoSan Juan, Puerto Rico

**Keywords:** CB_1_ receptors, signaling, endocannabinoid system, neuromodulation, Δ^9^-THC

## Abstract

The identification and cloning of the two major cannabinoid (CB_1_ and CB_2_) receptors together with the discovery of their endogenous ligands in the late 80s and early 90s, resulted in a major effort aimed at understanding the mechanisms and physiological roles of the endocannabinoid system (ECS). Due to its expression and localization in the central nervous system (CNS), the CB_1_ receptor together with its endogenous ligands (endocannabinoids (eCB)) and the enzymes involved in their synthesis and degradation, has been implicated in multiple pathophysiological events ranging from memory deficits to neurodegenerative disorders among others. In this review, we will provide a general overview of the ECS with emphasis on the CB_1_ receptor in health and disease. We will describe our current understanding of the complex aspects of receptor signaling and trafficking, including the non-canonical signaling pathways such as those mediated by β-arrestins within the context of functional selectivity and ligand bias. Finally, we will highlight some of the disorders in which CB_1_ receptors have been implicated. Significant knowledge has been achieved over the last 30 years. However, much more research is still needed to fully understand the complex roles of the ECS, particularly *in vivo* and to unlock its true potential as a source of therapeutic targets.

## Introduction

The endocannabinoid system (ECS) plays key modulatory roles during synaptic plasticity and homeostatic processes in the brain. Based on anecdotal evidence obtained from cannabis use, laboratory studies, and from emerging clinical work, modulation of the ECS has been proposed as a promising therapeutic target to treat numerous central nervous system (CNS) disorders including neurodegenerative diseases, epilepsy and cognitive deficits among others (Scotter et al., 2010; Fernández-Ruiz et al., 2011; Bilkei-Gorzo, 2012). However, the widespread expression and complex roles of several components of the ECS in excitatory and inhibitory transmission makes the development of such therapy highly challenging (Di Marzo, 2008). This review will explore some of the relationships between the cannabinoid (CB_1_ and CB_2_) receptors and their ligands with the nervous system in health and disease. We will introduce the two major receptors, focusing on the CB_1_ receptors due to their high expression levels in the CNS; their endogenous ligands or endocannabinoids (eCB) and some synthetic mimetics that activate and modulate their signaling; the signaling pathways that connect this receptor to processes inside the cell; and the role of the CB system in the normally functioning CNS and its alteration or therapeutic modulation in a variety of disease states.

## CB_1_ Receptors

**Figure 1:**
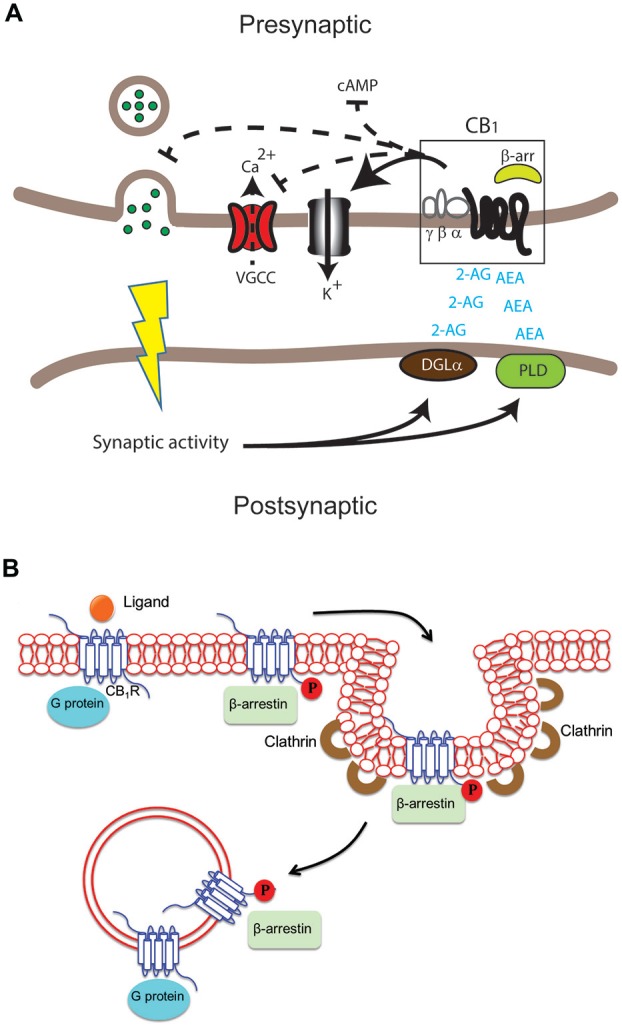
**Differential cannabinoid (CB) receptor signaling modalities can impact neuromodulation in health and disease in specific ways. (A)** Key enzymes such as diacylglycerol lipase (DGLα) and phospholipase D (PLD) produce the endogenous ligands arachidonylethanolamine (AEA) and 2-arachidonylglycerol (2-AG). These activate the cannabinoid 1 receptor (CB_1_) receptor in the central nervous system (CNS). The result can include modulation of adenylate cyclase activity to inhibit cAMP accumulation, voltage-gated calcium channels (VGCC), K+ channels and neurotransmitter release in presynaptic excitatory and inhibitory synapses. **(B)** Following activation of the CB_1_ receptor by ligand binding, signaling via G protein and/or β-arrestin may occur at the plasma membrane, in endocytic pits or in endosomes after internalization of the receptor. G proteins usually bind the unphosphorylated receptor while β-arrestin binds the receptor phosphorylated by G protein receptor kinases.

The CB_1_ receptor is one of the most abundant G protein-coupled receptors (GPCRs) in the CNS and is found in particularly high levels in the neocortex, hippocampus, basal ganglia, cerebellum and brainstem (Herkenham et al., 1991; Marsicano and Kuner, 2008). CB_1_ receptors are also found on peripheral nerve terminals and some extra-neural sites such as the testis, eye, vascular endothelium and spleen. Interestingly, CB_1_ receptors are highly enriched at presynaptic and axonal compartments, restricting their function to sites of synaptic activity (Straiker and Mackie, 2005; Wu et al., 2008). In addition to its location on the cell surface, intracellular localization of CB_1_ receptors has also been reported in heterologous systems and primary cultures (Leterrier et al., 2006; Rozenfeld, 2011). The CB_1_ receptor binds the main active ingredient of Cannabis sativa (marijuana), Δ^9^-tetrahydrocannabinol (Δ^9^-THC) and mediates most of the CNS effects of Δ^9^-THC (Zimmer et al., 1999). In addition, CB_1_ receptors bind synthetic cannabimimetic compounds such as CP55940, JWH-015, WIN55212-2 and the endogenous arachidonic acid derivatives arachidonylethanolamine (AEA) and 2-arachidonylglycerol (2-AG; see below; Howlett et al., 2002). Upon ligand binding and receptor activation, CB_1_ receptors are primarily coupled to pertussis toxin (PTX)-sensitive Gi/o type G proteins which leads to a rapid decrease in levels of cAMP by inhibiting adenylate cyclase activity (Figure 1A; Howlett et al., 2004). Coupling to other G proteins including Gs, albeit with low efficacy, can also stimulate adenylate cyclase (Glass and Felder, 1997; Glass and Northup, 1999; Varga et al., 2008; Bosier et al., 2010) though the extent of accumulation of cAMP is not necessarily a good indicator of G protein coupling (Eldeeb et al., 2016). Evidence of promiscuous coupling to different G proteins, signaling roles mediated by β-arrestins and signaling from intracellular compartments (Figure 1B) adds yet another level of complexity making these receptors, like other GPCRs, pluridimentional (Bosier et al., 2010). For our recent review on the multiple waves of receptor signaling see Nogueras-Ortiz and Yudowski (2016). CB_1_ receptors exhibit constitutive activity indicative of G protein activation in the absence of agonists and this could mediate their highly polarized localization to axonal and presynaptic compartments (Bouaboula et al., 1997; Nie and Lewis, 2001; Rozenfeld, 2011). The activity associated with this state is reversed by treatment with inverse agonists such as SR141716A (also called rimonabant). The model for GPCR activation has been adapted to include these multiple states (Perez and Karnik, 2005; Park et al., 2008) with distinguishing biochemical characteristics, including extent and selectivity of G protein coupling (Mukhopadhyay and Howlett, 2001; Kenakin, 2004). The recent crystallization of the CB_1_ receptor bound to the antagonist AM6538, should provide new opportunities for understanding the structure-function relationship of this receptor and help novel drug design (Hua et al., 2016).

## CB_2_ Receptors

The CB_2_ receptor exhibits a more defined pattern of expression in the brain than CB_1_ receptors, and is found predominantly in cells and tissues of the immune system (Klein, 2005; Mackie, 2006). In the CNS, CB_2_ receptor expression is associated with inflammation and it is primarily localized to microglia, resident macrophages of the CNS (Mackie, 2008; Palazuelos et al., 2009). This selective localization together with the modulatory effect of the CB_2_ receptor on microglia function is particularly relevant since microglial cells have a significant role in Alzheimer’s disease (AD) and other diseases associated with the basal ganglia (Ramírez et al., 2005; Sagredo et al., 2007; Fernández-Ruiz et al., 2011; Yeh et al., 2016). Interestingly, recent work also indicates that CB_2_ receptors expressed in neurons can control synaptic function and are involved in drug abuse and synaptic plasticity (Xi et al., 2011; Stempel et al., 2016). For example, the selective CB_2_ receptor agonist JWH133 inhibits dopaminergic firing from the ventral tegmental area and reduced cocaine self-administration (Zhang et al., 2016). Furthermore, neuronal CB_2_ receptors work independently from CB_1_ receptors to modulate inhibitory plasticity in the CA2/3 regions of the hippocampus and gamma oscillations *in vivo* (Stempel et al., 2016). We predict more regulatory roles will be identified for the CB_2_ receptors expressed in neurons.

## Endocannabinoids

eCBs are produced on demand with their synthesis typically triggered via increased intracellular Ca^2+^ at postsynaptic sites in response to sustained synaptic activity (Figure 1A; Chevaleyre et al., 2006; Mackie, 2006; Heifets and Castillo, 2009). Major eCBs are rapidly deactivated by reuptake mechanisms and degrading enzymes, including fatty acid amide hydrolase (FAAH) and monoacylglycerol lipase (MAGL; Howlett et al., 2004; Mechoulam and Parker, 2013). Among eCBs, the derivatives of arachidonic acid such as AEA and 2-AG are dominant and orthosteric (Pertwee, 2015). These ligands are agonists for CB_1_ and CB_2_ receptors but bind CB_1_ receptors with higher affinity (AEA Ki = 89 nM and 321 nM for CB_1_ and CB_2_ receptors respectively; 2-AG Ki = 472 nM and 1400 nM for CB_1_ and CB_2_ receptors respectively; Pertwee et al., 2010). More recently, allosteric eCBs have been identified, including pregnenolone and lipoxin A4 which can modulate CB_1_ receptor signaling with possible therapeutic value (Pamplona et al., 2012; Vallée et al., 2014; Pertwee, 2015). Further pharmacological characterization is still needed of orthosteric and allosteric modulators to clearly elucidate their physiological roles and modes of action. Nevertheless, the pharmacological manipulation of eCB levels or their actions by allosteric modulators could provide alternative opportunities to regulate the ECS. For a comprehensive review on eCBs see Fonseca et al. (2013).

## The Endocannabinoid System in the CNS

The ECS has emerged as one of the key regulatory mechanisms in the brain controlling multiple events such as mood, pain perception, learning and memory among others (Marsicano and Lutz, 2006; Kano et al., 2009). It is also thought to provide a neuroprotective role during traumatic brain injury (TBI) and may be part of the brain’s natural compensatory repair mechanism during neurodegeneration (Pryce et al., 2003; Klein, 2005; Campbell and Gowran, 2007; Bilkei-Gorzo, 2012). New roles for the ECS in drug abuse and dependence are identified almost continuously, further strengthening the relevance of this system not only during cannabis abuse but also other illicit drugs as well (Maldonado et al., 2006; Xi et al., 2011; Parsons and Hurd, 2015). In the CNS, eCBs act as retrograde messengers mediating feedback inhibition modulating synaptic plasticity (Howlett, 2005; Chevaleyre et al., 2006; Katona and Freund, 2012). Specifically, activation of the CB_1_ receptor leads to activation of inwardly rectifying K^+^ channel conductance, decreases in N-type and P/Q-type voltage-operated Ca^2+^ channel conductance and eCB production (Figure 1A; Mackie et al., 1995; Twitchell et al., 1997; Guo and Ikeda, 2004; Demuth and Molleman, 2006). This results in a decrease of neurotransmitter release at excitatory and inhibitory synapses leading to transient effects, as in depolarization-induced suppression of inhibition (DSI) and depolarization-induced suppression of excitation (DSE) or persistent effects as in long-term depression and potentiation (LTP/LTD) during synaptic plasticity (Wilson and Nicoll, 2001; Chevaleyre et al., 2006; Heifets and Castillo, 2009; Kano et al., 2009; Castillo et al., 2012; Soltesz et al., 2015; Maroso et al., 2016). These events make the ECS a key modulator of synaptic plasticity.

Prolonged exposure to CB_1_ receptor agonists results in rapid attenuation of behavioral responsiveness, termed tolerance, in human and animal models that has been attributed to both a decrease in the ability of the receptor to activate effector pathways (i.e., desensitization) and in the reduction in the number of cell surface-expressed receptors (i.e., internalization; Howlett et al., 2004; Martini et al., 2007). At the molecular level, the agonist-bound GPCR becomes a substrate for G protein coupled receptor kinases (GRKs); these kinases phosphorylate serine and/or threonine residues on GPCR cytoplasmic domains, which then become a high affinity target for β-arrestins (Jin et al., 1999; Delgado-Peraza et al., 2016). Binding of β-arrestins uncouples G-proteins and stimulates receptor internalization and β-arrestin mediated signaling (Jin et al., 1999; Roche et al., 1999).

Ligand induced receptor phosphorylation by GRKs can result in very specific and distinct phosphorylation profiles or “bar-codes” (Butcher et al., 2011; Liggett, 2011; Delgado-Peraza et al., 2016). These bar-codes are finely tuned and define which signaling cascades are activated, thus opening up a spectrum of possibilities frequently defined as functional selectivity or ligand bias (Liggett, 2011; Nobles et al., 2011; Prihandoko et al., 2016). However, careful consideration must be taken when interpreting results obtained from heterologous systems, particularly when signaling can be significantly affected (biased) by the different levels of protein expression across different cell types (Bosier et al., 2010; Atwood et al., 2011; Straiker et al., 2012).

Supporting the bar-code hypothesis and identifying the mechanisms and signaling cascades downstream from the CB_1_ receptor/β-arrestins, our recent data indicates that receptor and β-arrestin interaction and signaling cascades are dependent on specific phosphorylation sites controlled by unique GRKs (Delgado-Peraza et al., 2016). Mutation of the putative GRK sites from S426/S430 to alanines (rat sequence conserved in human) resulted in reduced β-arrestin 2 recruitment and receptor internalization, but enhanced interaction with β-arrestin 1 and increased β-arrestin 1 mediated signaling (Ahn et al., 2013; Delgado-Peraza et al., 2016). Replacement of series 426/430 to alanines renders the CB_1_ receptors biased towards β-arrestin 1 signaling and provides an ideal tool to probe the signaling pathways, mechanisms and roles of these cascades. β-arrestin mediated signaling from this biased receptor controls the activation of several cascades including ERK1/2, JNK1/2/3, CREB and P38α. It is important to note that these cascades have been previously linked to the activation of CB_1_ receptors, but not all to β-arrestins (Rueda et al., 2000; Derkinderen et al., 2001; Hart et al., 2004). Activation of these cascades by CB_1_ receptors and β-arrestins resulted in the regulation of gene expression and protein synthesis (Delgado-Peraza et al., 2016).

Elucidating the physiological roles of β-arrestins may foster the development of pathway-selective or “biased ligands” with greater therapeutic benefit. Investigating signaling from biased CB_1_ receptors such as S426A/S430A and the DRY mutant (Asp-Arg-Tyr) together with the identification of biased ligands and the crystal structure of CB_1_ receptors should provide important tools to elucidate the mechanisms and roles of CB_1_ receptor signaling (Gyombolai et al., 2015; Delgado-Peraza et al., 2016; Hua et al., 2016).

The subcellular localization and trafficking of CB_1_ receptors is highly dynamic, with significant effects on receptor signaling (Leterrier et al., 2004; Brailoiu et al., 2011; Rozenfeld, 2011; Dudok et al., 2015). CB_1_-G protein mediated signaling occurs at the cell surface and at intracellular compartments (Rozenfeld and Devi, 2008; Brailoiu et al., 2014). At the cell surface, CB_1_ receptor ligands modulate the interaction between receptors and β-arrestin as a mechanism to influence β-arrestin mediated signaling (Flores-Otero et al., 2014). This interaction is initiated at the plasma membrane and can continue into intracellular compartments (Delgado-Peraza et al., 2016). Interestingly, these location-specific signaling events appear to be widespread among several GPCRs. For example, the LH receptor, β2 adrenergic receptor and the CB_2_ receptor can signal from intracellular compartments either by β-arrestins or G proteins via a “super-complex” ultimately resulting in three different spatio-temporal signaling waves (Brailoiu et al., 2014; Irannejad and von Zastrow, 2014; Lyga et al., 2016; Nogueras-Ortiz and Yudowski, 2016; Thomsen et al., 2016). Constitutive activation also plays a role in their trafficking (Leterrier et al., 2006; McDonald et al., 2007). CB_1_ receptor location and trafficking are highly dynamic events that are intimately intertwined with their signaling (Dudok et al., 2015). What is the role and relevance of this compartment selective signaling event? Considering the restrictive location of CB_1_ receptors to presynaptic sites, a possible role could be the local modulation of gene and protein expression after chronic receptor activation. Where do these intracellularly active receptors go and when do they stop signaling are intriguing questions that should provide clues to their physiological roles.

## Δ^9^-THC

The cannabis plant contains more than 60 different active synthetic ligands for CB1/2 (CBs) with Δ^9^-THC being the major psychoactive molecule among them (Brenneisen, 2007). Exposure to Δ^9^-THC leads to pleiotropic and sometimes paradoxical effects in humans including analgesic responses, relaxation, dysphoria, tolerance and dependence (Mechoulam and Parker, 2013). Most of these effects are blocked with SR141716, a selective blocker of CB_1_ receptors (Huestis et al., 2001). In rodents, repetitive administration of Δ^9^-THC results not only in tolerance but characteristically in a “tetrad” response which includes antinociception, hypothermia, hypoactivity and catalepsy (Little et al., 1988; Fride et al., 2006; Nguyen et al., 2012). However, lack of behavioral sensitization has also been described in mice chronically exposed to Δ^9^-THC (Varvel et al., 2007). At the molecular level, Δ^9^-THC acts as a partial agonist of the CB_1_ receptor, at the G protein level and as a potent activator of β-arrestin 2 recruitment and signaling in heterologous systems (Pertwee et al., 2010; Laprairie et al., 2014, 2016). Perhaps the complex behavioral responses to Δ^9^-THC could be mediated by the selective activation of these different signaling cascades. Interestingly, β-arrestins mediate some of the behaviors associated with long-term exposure to Δ^9^-THC (Breivogel et al., 2008; Wu et al., 2008). β-arrestin 2 KO mice display enhanced antinociceptive response to acute Δ^9^-THC and a decrease in tolerance, indicating the relevance of classical roles of β-arrestin (i.e., receptor desensitization) during G protein signaling (Nguyen et al., 2012). However, recent work on β-arrestin 1 KO mice indicates divergent roles of β-arrestin 1/2 and proposed that β-arrestin 1 regulates receptor sensitivity in an agonist dependent manner, with no significant effects regulating CB tolerance (Breivogel and Vaghela, 2015). Interestingly, our work and others also suggest β-arrestin 1 as the “signaling” arrestin for CB_1_ receptor. This divergence could be exploited to design compounds that are biased towards G protein signaling with less receptor desensitization and decreased tolerance as recently demonstrated for pain modulation with the mu opioid receptor (Manglik et al., 2016).

## CB_1_ Receptors in Disease

CB_1_ receptors are indicated in many disorders that impact the CNS including several neurodegenerative disorders such as Huntington’s disease (HD), multiple sclerosis (MS) and AD (Fernández-Ruiz et al., 2011; Di Marzo et al., 2014).

## Multiple Sclerosis

MS is a major immune-related neurodegenerative disease characterized by demyelinization with axonal and neuronal loss. Several clinical trials present positive effects of either cannabis, Δ^9^-THC or other CB agonist on spasticity, spasms and pain among other signs of MS (Croxford, 2003; Pertwee, 2007; Rog, 2010; Notcutt et al., 2012). Use of Sativex® (Nabiximol) an oromucosal spray of cannabis extract containing fixed concentrations of Δ^9^-THC and cannabidiol (CBD), results in symptomatic improvement in patients with MS. There is a reduction in motor dysfunction and pain, observed in meta-analysis of several clinical studies. However, an increased incidence of non-serious side effects was also reported (Wade et al., 2010; Otero-Romero et al., 2016). Importantly, a review by the National Institute for Health and Care Excellence in the United Kingdom, recommended against the use of Sativex® to treat spasticity in people with MS because it is not a cost effective treatment (Multiple sclerosis in adults: management | 1-recommendations | Guidance and guidelines | NICE, 2014). For a recent and comprehensive analysis of clinical studies see the work of Otero-Romero et al. (2016).

At the molecular level, these improvements are generally linked to the activation of both CB_1_ receptors and CB_2_ receptors by agonist, resulting in their dual anti-inflammatory and neuroprotective effects throughout the CNS (Baker et al., 2000; Maresz et al., 2007). These effects include up-regulation of prosurvival molecules such as interleukines in astroglia, and the reduction of cytotoxic factors such as nitric oxide, reactive oxygen species and proinflammatory cytokines in microglia (Fernández-Ruiz et al., 2011). The precise mechanisms by which receptors exert their neuroprotective activity might include activation of phosphatidylinositol 3-kinase/mammalian target of rapamycin complex 1 (mTOR1) pathway and brain-derived neurotrophic factor (BDNF; Ozaita et al., 2007; Blázquez et al., 2015).

Consistent with the clinical data, using synthetic CBs lead to a reduction in inflammation and neuropathic pain in the Experimental Autoimmune Encephalomyelitis (EAE) mouse model (Pryce et al., 2003; Maresz et al., 2007; Fu and Taylor, 2015). Similar results were observed with systemic treatment with the agonists, WIN55212-2, ACEA and JWH-015 of mice with established Theiler’s Murine Encephalomyelitis Virus-induced Demyelinating Disease, a mouse model of chronic progressive MS. Mouse motor function was improved by modulating microglia and lymphocyte infiltration into the spinal cord (Arévalo-Martín et al., 2003). In contrast, when an inverse agonist of the CB_1_ receptor (SR141716A) was applied, the EAE was worsened likely by releasing pro-inflammatory cytokines in the mouse brain and spinal cord (Saito et al., 2012). Underlying the role of CB_1_ receptors during neuromodulation and inflammation, work on CB_1_ receptor^−/−^ mice suggest that these animals are more susceptible to neurotoxicity and damage when compared to wild-type mice (Jackson et al., 2005; Pertwee, 2007). Taken together these results suggest that in MS, the neuroprotective roles of CB_1_ and CB_2_ receptors might be impaired and their enhancement could provide new therapeutic approaches. For a comprehensive review of the literature of MS from model systems to clinical studies see Pertwee (2007) and Rog (2010).

## Huntington’s Disease

Dysregulation of the ECS is also reported in experimental models and patients with HD. The CB_1_ receptor expression is reduced, at least somewhat (e.g., 27% decrease in the striatum of the CB_1_ receptor mRNA), prior to symptoms of neurodegenerative HD in mice (McCaw et al., 2004). Losing the CB_1_ receptor expression decreases motor performance and increases the amount of aggregates in the striatum of HD mice (Mievis et al., 2011). Major loss of CB_1_ receptors is also reported in patients with HD (Glass et al., 2000). Interestingly, activation of the CB_1_ receptor may help reduce the progression of HD. For example, preclinical evidence suggested the use of CBs such as Sativex® for neuroprotection in patients with progressive neurodegenerative conditions like HD (Valdeolivas et al., 2012). Furthermore, selected receptor agonists have neuroprotective potential in a cell culture model of HD (Scotter et al., 2010; Laprairie et al., 2016). Interestingly, ligands biased to β-arrestin mediated signaling such as Δ^9^-THC, reduced cellular function and viability in these models, suggesting a potential pharmacological profile for therapeutic agonists (Laprairie et al., 2014, 2016). These events are mediated in part by the activation of Gα_i/o_ mediated pathways and might limit glutamate release from cortical neurons and GABA from striatal medium spiny neurons (Dowie et al., 2010; Laprairie et al., 2016). Results obtained investigating the R6/2 mouse model of HD, indicate that CB_1_ receptor activation parallels *BDNF* expression leading to neuroprotection (Blázquez et al., 2015). In general, the *in vivo* and *in vitro* data suggest that CB agonist with specific pharmacological profiles (biased towards *BDNF* upregulation and release) could be developed to treat or ameliorate HD.

## Alzheimer’s Disease

CB_1_ receptors have also been the focus of intense research as a potential target in AD. This work has been performed *in vitro*, animal models and *post-mortem* samples. Changes in the expression levels of several components of the ECS in *post-mortem* samples from AD patients have been identified, although their role in the pathophysiology of the disorder is still unknown. For example, CB_1_ receptors in hippocampus from patients with AD were not different from aged-matched controls. However, the levels of MAGLs, the degradative enzyme of 2-AG, were reduced at their site of action in these patients, suggesting an altered eCB signaling and architecture (Mulder et al., 2011). Limited positive behavioral results have been observed in small clinical trials and pilot studies using analogs of Δ^9^-THC (Aso and Ferrer, 2014). Analysis of the studies and trials available, suggest significant benefits from synthetic CBs on some of the behavioral and psychological symptoms of dementia (Liu et al., 2015). However, these conclusions were based on short and limited studies; further work will be needed to assess the safety and efficacy of CBs in AD. In experimental models of AD, several findings indicate that the activation of both CB_1_ receptors and CB_2_ receptors might have beneficial effects mainly through neuroprotection against Aβ toxicity as previously noted for other neurodegenerative disorders. For example, by crossbreeding the AD mouse model (APP23) with the CB_1_ receptor-deficient mouse, enhanced cognitive impairment was observed while presenting a reduced amyloid deposition (Stumm et al., 2013). Tau protein phosphorylation is also reduced by CBD in PC12 cells, providing a different neuroprotective mechanism during AD (Esposito et al., 2006). Since CB_1_ receptors are not likely directly activated by CBD, the impact on Tau phosphorylation may be via the antioxidant effect of CBD or perhaps as a CB receptor independent effect. A reduction in harmful β-amyloid peptide and tau phosphorylation, while promoting intrinsic CNS repair mechanisms may take place consecutively due to activation of the immune and CNS CB system in AD (Aso and Ferrer, 2014). For example, recent work on the TREM2 receptor in microglia, where CB_2_ receptors are expressed and control cellular responses, also provides an immune related mechanism to control AD (Yeh et al., 2016).

Aging is a major risk factor for neurodegenerative diseases and neuronal progenitor cell proliferation is greatly reduced in the process. Remarkably, CBs can stimulate embryonic and adult neurogenesis (Jiang et al., 2005; Trazzi et al., 2010). Axonal guidance, cell migration, synapse formation and cell survival are also modulated during development. Dysregulation of these processes during development and aging could significantly contribute to multiple disorders of the CNS. For an extensive and thorough review of this topic see the work of Di Marzo et al. (2014) and Maccarrone et al. (2014).

## Traumatic Brain Injury

There is good agreement that the CB_1/2_ receptors are involved in TBI and that 2-AG increases after TBI in animal models (Panikashvili et al., 2001; Mechoulam and Shohami, 2007). There is an “on-demand” signal to generate eCB following TBI that can decrease brain edema and inflammation (Shohami et al., 2011; Gruenbaum et al., 2016). These events may be neuroprotective and prevent excitotoxicity, inhibit inflammatory cytokine production and augment stem cell migration and differentiation. Furthermore, CB_1_ receptor and CB_2_ receptor antagonists prevent drug-induced neuroprotection in a mouse mode of TBl (Lopez-Rodriguez et al., 2015). However, as indicated previously for other disorders, limited clinical data is available to support efficacy and safety of CBs during TBI (Gruenbaum et al., 2016).

## Future Studies

The modulation of the ECS has great therapeutic potential in many neuropsychiatric and neurodegenerative disorders. Our understanding of the *in vivo* and *in vitro* pharmacology of the CB_1_ receptors and CB_2_ receptors has significantly increased over the last decades, with new insights into the pathways controlled and the roles of these receptors, enzymes and ligands emerging regularly in the literature. However, this knowledge has not made a complete transition into drug development yet. Complicating this progression, is the mounting anecdotal evidence obtained from cannabis use, which contains over 60 CBs plus other relevant compounds at different concentrations. This variability, together with limited information from clinical trials makes it difficult to scientifically assess the multiple claims associated with cannabis use. Careful investigation of defined molecular entities, in randomized double blind, placebo controlled and multicentric studies should be implemented to clearly move the field forward. At the same time, further work should be performed utilizing cellular and animal models to clearly identify the desired mechanisms and signaling pathways to be therapeutically targeted.

## Author Contributions

DAK and GAY wrote and revised this article.

## Conflict of Interest Statement

The authors declare that the research was conducted in the absence of any commercial or financial relationships that could be construed as a potential conflict of interest.

## Abbreviations

AD: Alzheimer’s Disease
AEA: arachidonylethanolamine
2-AG: 2-arachidonoylglycerol
CB_1_ receptors: cannabinoid 1 receptor
CB_2_ receptor: cannabinoid 2 receptor
CBD: cannabidiol
CBs: synthetic ligands for CB1/2
CNS: central nervous system
eCBs: endocannabinoids
GPCR: G protein-coupled receptor
HD: Huntington’s disease
MS: multiple sclerosis
TBI: traumatic brain injury.
